# Being silenced, loneliness and being heard: understanding pathways to intimate partner violence & abuse in young adults. a mixed-methods study

**DOI:** 10.1186/s12889-022-13990-4

**Published:** 2022-08-17

**Authors:** Maria Barnes, Eszter Szilassy, Annie Herbert, Jon Heron, Gene Feder, Abigail Fraser, Laura D. Howe, Christine Barter

**Affiliations:** 1 Department of Population Health Sciences, University of Bristol, Canynge Hall, Whatley Road, Bristol, BS8 2PS UK; 2 University of Central Lancashire, Preston, UK

**Keywords:** Risk, Loneliness, Vulnerabilities, Intimate partner violence, Young adult, Qualitative, Mixed methods, ALSPAC

## Abstract

**Background:**

International research shows the significance and impact of intimate partner violence and abuse (IPVA) as a public health issue for young adults. There is a lack of qualitative research exploring pathways to IPVA.

**Methods:**

The current mixed-methods study used qualitative interviews and analysis of longitudinal cohort data, to explore experiences of pathways to IPVA. Semi-structured Interviews alongside Life History Calendars were undertaken to explore 17 young women’s (19–25 years) experiences and perceptions of pathways to IPVA in their relationships. Thematic analysis was undertaken.

Based on themes identified in the qualitative analysis, quantitative analysis was conducted in data from 2127 female and 1145 male participants of the Avon Longitudinal Study of Parents and Children (ALSPAC) birth cohort study. We fitted regression models to assess the association of child maltreatment, parental domestic violence, and peer-to-peer victimisation, by age 12, with loneliness during adolescence (ages 13–14), and the association of loneliness during adolescence with IPVA (age 18–21). Mediation analysis estimated the direct effects of maltreatment on IPVA, and indirect effects through loneliness.

**Findings:**

All women interviewed experienced at least one type of maltreatment, parental domestic violence, or bullying during childhood. Nearly all experienced IPVA and most had been multi-victimised. Findings indicated a circular pathway: early trauma led to isolation and loneliness, negative labelling and being silenced through negative responses to help seeking, leading to increased experiences of loneliness and intensifying vulnerability to further violence and abuse in young adulthood. The pathway was compounded by intersectionality. Potential ways to break this cycle of loneliness included being heard and supported, especially by teachers.

Quantitative analysis confirmed an association between child maltreatment and loneliness in adolescence, and an association between loneliness in adolescence and experience of IPVA in young adult relationships.

**Conclusion:**

It is likely that negative labelling and loneliness mediate pathways to IPVA, especially among more disadvantaged young women. The impact of early maltreatment on young people’s wellbeing and own relationships is compounded by disadvantage, disability and ethnicity. Participants’ resilience was enabled by support in the community.

**Supplementary Information:**

The online version contains supplementary material available at 10.1186/s12889-022-13990-4.

## Background

International research has shown the significance and impact of intimate partner violence and abuse (IPVA) as a public health issue in young adults [1]. Prevalence rates for IPVA in young adults vary considerably depending on the populations sampled, definitions used and the forms of IPVA included [2], although rates are considered high, with reported prevalence of up to 97% for emotional and psychological violence [3]. A range of demographic, mental health, and behavioural factors are associated with increased prevalence of IPVA victimisation or perpetration [4]. Evidence has consistently shown that young people with a history of familial domestic violence or child abuse are at greater risk of IPVA [2, 5, 6]. Other research has highlighted a range of wider factors associated with the risk of IPVA including: bullying, social norms, gendered attitudes and beliefs, mental health problems and drug use [7, 8]. Feelings of loneliness and isolation are one of the consistent consequences reported by women in abusive intimate relationship and children who have been sexually abused [9]. Importantly, not all young people who experience familial violence go on to experience IPVA [10, 11]. Protective factors which have received the strongest empirical support and demonstrated significant additive and/or buffering effects in longitudinal studies are self-regulation, family support, school support, and peer support [12].

There is a broad consensus for the need to identify the complex pathways to IPVA across different countries and populations, which consider socio-ecological factors to inform primary prevention and interventions [1, 2, 8]. To do this we need to better understand the pathways to violence and abuse through adopting an ecological perspective. Ecological perspectives emphasise the complexity and bi-directional influence of the interactions that young people experience at four levels: the individual, family and relationships, community, and wider societal context and note that these interactions can protect or increase vulnerability to IPVA [1]_._

However, most research in this area still focuses on investigating the links at personal and family levels, whilst recognising more work is needed to understand how the broader community and societal levels can contribute to either sustaining or preventing domestic violence [13]. Hamby [14] summarises that research which identifies the importance of community level factors remains largely absent from the literature on violence and adversity. In contrast to most current prevention approaches that focus on risks or how to avoid risk/violence, a strengths-based approach to addressing violence, including IPVA, is posited as the most promising way to direct prevention and intervention efforts [15]. Strengths-based frameworks challenge us to think about what individuals, groups or communities are striving for or moving towards in their lives, and the assets or protective factors that help them achieve this.

The complexity of pathways leading to IPVA suggest that qualitative research is necessary to support a more nuanced understanding of young adults’ experiences and perceptions of these pathways and what helps them overcome trauma and disadvantage. However, most research in this area is quantitative (e.g [16, 17].) with only a small number of qualitative studies exploring participants own understanding and perception around pathways to intergenerational IPVA [18, 19] and none specific to UK populations. A mixed-methods approach synthesising findings would strengthen and expand our knowledge of these pathways.

The ability to support young adults also depends on being able to identify those in need. Disclosure of maltreatment has been suggested as an ongoing process unfolding over time, where positive and negative feedback loops are possible depending upon the response received by the young person disclosing [20]. Consequences can be both positive and negative, impacting on the young person's subsequent help seeking attempts [21, 22].

Much of the focus in the literature has been on disclosure of childhood sexual abuse, although many studies have reported that sexual abuse often occurs alongside other forms of abuse, for example neglect [23, 24]. There is also a need to consider disparities in disclosing between different groups of children and young adults and types of abuse, including multi-victimisation where multiple forms of abuse have been experienced [25]. Finkelhor (2007 [26]) considered that weaknesses in previous studies on child victimisation focussed on the contribution of single victimisation experiences to mental health problems and often failed to identify chronically—or poly-victimised—children and how this can affect them in more traumatic and less reversible ways [27]. Poly-victimisation is a strong predictor of trauma symptoms in children [26], worse psychological impairment in adolescence [28], along with other multiple and adverse consequences [5, 29]. The designation and analysis of poly-victims can aid in assessing the cumulative impact of, and better understanding of victimisation trauma [30–32].

Some research has specifically investigated the intersection of child maltreatment and IPV, which often co-occurs and is underscored by community violence and social and structural factors that add more stress and trauma to lives [33]. It is posited that the response to this should be large-scale public health strategies emphasising primary prevention and focussing on strength-based approaches to build resiliency [33].

As with the strengths-based approach, focussing on what helps, not hinders, children and young people from disclosing maltreatment would also be beneficial [34].

The current Young Adults Relationships and Health Study (The YARAH study—http://www.bristol.ac.uk/primaryhealthcare/researchthemes/yarah-study/link) addresses the gaps in qualitative empirical evidence by exploring the understandings and perspectives of a sample of young women who had experienced IPVA (more than half who had been multi-victimised) about the pathways to IPVA and highlights factors that helped participants’ resilience. It also builds on, and extends the evidence of, the dynamic, dialogic process of disclosure and help-seeking in young people who have experienced domestic violence and abuse (DVA) and maltreatment in their family of origin. By viewing the data within an ecological framework, we also highlight the importance of responses at all levels and interactions for the young person seeking help. To triangulate the findings from our qualitative interviews, we used quantitative data from a longitudinal cohort study to explore a key pathway identified in thematic analysis of our interview data: from early childhood trauma to IPVA, through loneliness.

**Table:** 

**Ecological Definition**
Individual level: characteristics of child, including inherited genetic and biological factors, age, disability or health, characteristics of child’s parents Relationship: child’s or young person’s interactions with others in the context of close relationships (family, friends, peers and intimate partners) Community: settings and institutions in which child’s relationships and interactions take place (neighbourhood, schools, residential units, workplaces and criminal justice agencies), Societal: laws, cultural and belief systems, social inequalities and political issues, such as gender inequality, social exclusion and poverty

We used the definition of ‘domestic abuse’ as defined in the UK Domestic Abuse Act (Legislation.gov.uk 2021) whereby behaviour is “abusive” if it consists of any of the following: physical or sexual abuse; violent or threatening behaviour; controlling or coercive behaviour; economic abuse; psychological, emotional or other abuse; and it does not matter whether the behaviour consists of a single incident or a course of conduct. IPVA is also domestic abuse and consists of the same behaviours but refers specifically to abuse that occurs within an intimate relationship only, and not the wider family.

## Methods

### Qualitative methods

Qualitative, face-to-face semi-structured interviews using Life History Calendars (LHCs [35];) were used to recall and explore respondents’ experiences in various domains (such as family, peers, schools, social medial) from across their life-course.

Research on autobiographic memory and survey methodologies has consistently found that the longer the reporting period between events and interview, the greater the propensity for underreporting and inaccuracy by participants [36, 37]. The LHC method combines a visual calendar with a semi-structured interview schedule to help respondents gain better access to and encourage the use of memory cues, in recalling patterns of past events.

The interview topic areas were developed from the literature, research team expertise and public and patient involvement (PPI) panel. Topics included asking young people about: experiences of family; education; friendships; intimate relationships; mental and physical health; wellbeing and actions e.g. help-seeking. Within each area, probe questions were prepared that allowed for expansion of the topic.

#### Sample

Eligible participants were those between 18–25 years old who had experience of domestic violence in their family of origin and/or intimate partner violence and abuse. Due to the nature of the research questions, ethical considerations guided recruitment to be only with young adults who had access to support—whether through frontline multi-agency organisations providing support/housing/advice, online support forums (such as for LGBT + community) or counselling services. Frontline service providers passed on information about the study to eligible participants where it was deemed safe to do so.

To ensure a range of participants were recruited, information about the study was also cascaded through various LGBT + networks online, university media (targeted at those with access to university mental health support), and the YARAH study website.

Written consent was obtained prior to the interviews which were audio-taped. All interviews were carried out by the same researcher (MB) between August 2019 and August 2020. Interviews lasted between one and three hours. Seventeen interviews were carried out face-to-face in participants’ homes, a private room in frontline organisation offices or the university. In March 2020, due to the Covid-19 pandemic, four interviews were carried out via a confidential video platform supported by the University. Case notes were written up after each interview.

#### Analysis

The interview data were transcribed verbatim. Data collection and analysis occurred concurrently and iteratively, cross-sectionally and in case-studies according to the constant comparison methods of grounded theory [38]. Thematic analysis was used to develop and refine clusters of themes from the data. LHC data were used as part of the participants’ case-studies to cross-check information and give a life-course narrative perspective.

Data relating to the first four interviews were analysed by detailed scrutiny of the transcripts which were then coded with the aid of NViVo12 software. A coding comparison exercise then took place with other members of the qualitative research team where codes were refined and the framework developed to code further transcripts within the sample and across sets, i.e. young people recruited through frontline organisations and young people recruited through other avenues.

Themes were developed from the data both deductively from the research questions (e.g. Help-seeking) and inductively (e.g. Loneliness/Isolation). Summaries of the case-studies of each participant were also made and considered for similarities and differences. Data saturation was reached in the themes presented. All names and identifying features have been changed in the descriptive quotes used.

A PPI panel informed the research, giving their perspective from their lived experience of early years maltreatment and/or IPVA. Their feedback contributed to the topic guide, recruitment process and analysis of data.

### Quantitative methods

Our quantitative analyses were designed to triangulate the key theme identified in our qualitative analysis, namely the importance of loneliness in the pathway to IPVA.

#### Sample

Data were from the Avon Longitudinal Study of Parents and Children (ALSPAC). Pregnant women resident in Avon, UK with expected dates of delivery 1st April 1991 to 31st December 1992 were invited to take part in the study. The initial number of pregnancies enrolled is 14,541. When the oldest children were approximately 7 years of age, an attempt was made to bolster the initial sample with eligible cases who had failed to join the study originally, resulting in an additional 913 children being enrolled. Information has been regularly collected since enrolment until present. Study data were collected and managed using REDCap electronic data capture tools hosted at University of Bristol [39]. More information on both the mothers and their offspring is available in published cohort profiles [40–42].The study website contains details of all the data that is available through a fully searchable data dictionary and variable search tool. (http://www.bristol.ac.uk/alspac/researchers/our-data/).

Different types of child maltreatment by the age of 12 years were reported by parents of participants when the child was 8 months, and 1, 2, 3, 5, 6, 7, 8, 9, and 11 years old, and the participants themselves at ages 8, 9, 12, and (retrospectively at) 23 years old [43]. From these questions, we derived binary measures of emotional abuse, physical abuse, sexual abuse, emotional neglect, domestic violence, and peer victimisation (bullying) occurring at any time-point (further details in Supplementary Materials), and combined these into a single binary measure of child maltreatment that was coded as 1 if participants reported one or more of emotional, physical or sexual abuse, emotional neglect, domestic violence, or peer victimisation, and 0 if they reported none of these forms of maltreatment.

To reflect the ecological framework as much as possible within the limits of the existing data in ALSPAC, we attempted to capture loneliness in different domains of life. Loneliness at school was defined as either strongly agreeing or agreeing (versus disagreeing or strongly agreeing) with the statement ‘school is a place I feel lonely’ in a self-completed questionnaire at age 14. Loneliness within the peer group was defined as reporting that ‘my friends understand me’ not often or not at all (versus sometimes or most of the time) at age 13. A measure of overall loneliness was taken from the Moods and Feelings Questionnaire administered at age 13 years, which asks whether the respondent felt lonely in the past two weeks; loneliness was defined as reporting ‘true’ versus ‘sometimes’ or ‘not at all’. No measure of loneliness within the family environment is available in ALSPAC.

At age 21, ALSPAC participants were asked about IPVA. For example, how often a partner had ‘Told you who you could see and where you could go and/or regularly checked what you were doing and where you were (by phone or text)?’, to which one could respond ‘never’, ‘once’, ‘a few times’, or ‘often’, and whether this occurred prior to turning 18, after turning 18, or at both time-points. The questionnaire also stated “By 'partner', we mean anyone you have ever been out with or had a relationship with, long-term or short-term (including 'one night stands').’ These questions been previously developed based on previous UK and European questionnaires and the PROVIDE questionnaire [44, 45] (and are described in full in a report of their psychometric properties) [46]. The questions are provided in Supplementary Box S1. As in previous work, we considered any response of at least ‘once’, to any of the eight questions as exposure to IPVA, because the header of the questionnaire was *‘Intimate Partner Violence’*, likely raising the threshold of severity for reporting certain behaviours, and because for participants who answered at least ‘once’ to any of the questions, negative impact was reported by 75–99% [8]. To ensure temporality of our exposure, mediator, and outcome, we only used data on IPVA exposure between ages 18–21 years.

The sex of participants was determined through obstetric records; maternal age at delivery was collected by fieldworkers visiting wards; maternal education was self-reported in a questionnaire during pregnancy, and categorized into four groups: degree or higher, A-levels, O-levels, or CSEs, vocational or no qualifications; whether the child lives in a single parent household is reported by the mother during pregnancy.

We used logistic regression to estimate the association of child maltreatment with each measure of loneliness, and the associations of child maltreatment and each measure of loneliness with IPVA, with and without adjustment for covariates (at the time of the ALSPAC offspring’s birth: mother’s age, mother’s highest educational qualification, parity, and whether a single parent or not). To estimate the degree to which loneliness mediates the association between child maltreatment and IPVA, we used the ‘paramed’ command in Stata (version 16.1) to estimate direct effects (i.e. the association between maltreatment and IPVA after accounting for loneliness) [47]. As the data we used were collected over a 23 year period, there are missing data due to loss to follow-up and non-completion of all relevant questionnaires. To maximise power and reduce the chance of selection bias, we used multivariate multiple imputation to impute missing values. Participants were included in our analysis if they had data on maltreatment and/or IPVA. We imputed 20 datasets using the ‘ice’ command in Stata – all analyses were carried out in each separate imputed dataset, and results pooled using Rubin’s rules. All imputation and analyses were conducted separately in women and men, because of sex differences in the prevalence and impact of IPVA [8, 48]. All analyses were carried out in Stata/MP 16; relevant code scripts are available on https://github.com/pachucasunrise/IPVA_loneliness.

#### A note on mixed methods

There are different ways of mixing methods and a variety of classifications of types (see [49]). It has been suggested that the important distinction is where in the research process the methods are mixed [50]. For the current study, the exploratory, mixed methods took place in the analysis phase. Phase I of the analysis was qualitatively led and initial findings around loneliness led to discussions within the research team about the quantitative secondary data including: how prevalent is loneliness in young people in general in the ALSPAC sample? Does IPVA lead to increased risks of loneliness or loneliness lead to increased risks of IPVA?

Further analysis of qualitative data demonstrated loneliness in participants was established in early years due to maltreatment and the ‘Loop of Loneliness’ was hypothesised (i.e. that maltreatment led to loneliness, leading to further re-victimisation). This led to further discussion in the research team on the research question: To what extent does loneliness mediate the path from maltreatment to later IPVA?

Following these discussions informed by the qualitative findings, we carried out a quantitative analysis of the secondary data, which established: links between child maltreatment and loneliness; loneliness and IPVA; and that loneliness might mediate the pathway from maltreatment to IPVA.

Integration of findings from the qualitative and quantitative analyses suggests the Loop of Loneliness as a plausible mechanism from maltreatment to IPVA. The inferences drawn were that pre-existing vulnerabilities and poly-victimisation need to be addressed by services as early as possible in this loop, in order to be effective, and that there are opportunities to mediate the impact of loneliness at the public health and community level.

## Findings

### Qualitative findings

Seventeen young women between 18–25 years old were interviewed. (Table 1).

**Table 1:** Characteristics of Young Women, Background and Maltreatment

	***n* = 17**
**Ethnicity**	
-White British	12
-Black British/dual	2
-South Asian British/dual	2
-White Eastern Europe	1
**Sexuality**	
-Heterosexual	12
-Bisexual	2
-Pansexual	2
-Not know	1
**Family of Origin**	
-Addictions	5
-Antisocial behaviour	8
-Health problems [mostly severe mental health]	10
**DV in Family of Origin**	**13**
-Physical	8
-Sexual	5
-Emotional	7
-Control	4
-Neglect	7
**Bullying by Peers**	14
**Other Sexual Assault**	6
**IPVA**	16
**IPVA**	**16**
-Emotional	15
-Control [incl. via children and abuser’s family]	14
-Physical	9
-Sexual	9
-Financial	5
-Gaslight	3

Survey information on socioeconomic classification was not taken

All participants, except one, had experienced IPVA in at least one relationship. None were currently in an abusive relationship. Just over half (13/17) had experienced DVA in their family of origin and the same proportion (13/17) had experienced both DVA in their family of origin and IPVA.

Whilst participants were recruited if they reported DVA (including IPVA), it became clear that a large majority had also experienced at least one type of maltreatment prior to their abusive intimate relationship. Maltreatment included: being aware of DVA within their family of origin, sexual abuse or grooming by adults (not parents) or peers, neglect by family, or severe bullying by peers.

More than half of the participants were recruited through frontline services and had a long and complex history of negative life events and had attempted help-seeking over the life course and prior to their IPVA.

For brevity in this article all forms of abuse and victimisation that is not IPVA will be defined under the umbrella term ‘maltreatment’.

The interview data showed that most participants had experienced multiple forms of victimization. The majority of participants’ accounts were dominated by narratives of not being heard or believed when they attempted to talk about their experiences of maltreatment or home-life and how negative labelling and disbelief by family, peers, community and society caused intense feelings of loneliness and isolation. Feeling lonely and isolated led to more vulnerability to further maltreatment, including abusive intimate relationships. Participants accounts also showed ways to ameliorate the impact of loneliness and support their wellbeing. Building on these findings two main themes were developed from the data: 1) Isolation and Loneliness with the subtheme of being Silenced through Labelling and Disbelief; and 2) Ways out of Loneliness: Finding a Voice with subthemes of Grandmother; Finding Good Friends and Taking Control; and Education and Achievement.

**Fig. 1:**
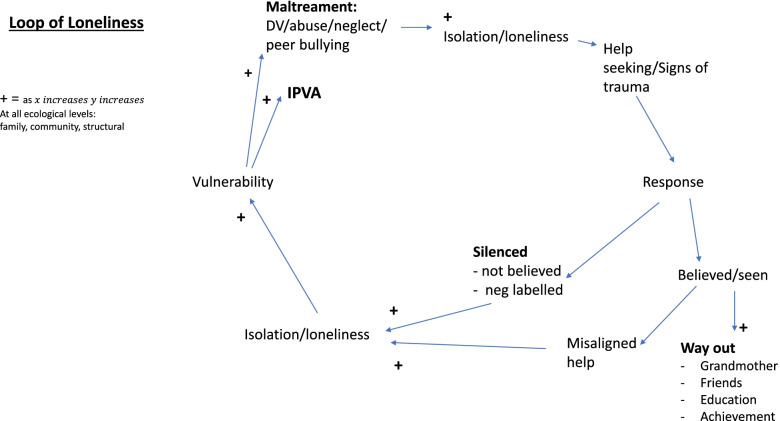
Loop of loneliness

The first section will describe findings on how help-seeking was responded to at all ecological levels by participants family, peers, community and society; and how it impacted on the participants’, well-being, identity and consequent help-seeking behaviours. The second section gives findings on what helped alleviate the impact at the different ecological levels. The findings can most easily be visualised as a loop as shown in Fig. 1.

#### Relationship level (family, partner)

Loneliness can be a possible consequence of the shame young people exposed to childhood violence often feel, which can then increase the risk of poor mental and physical health [51]. Feeling alone, loneliness, isolated or an outsider was mentioned by all but one of the participants across the range of socio-economic groups and backgrounds. The young women who had been multi-victimised had usually experienced early years maltreatment. As a result, loneliness often started in the earliest years due to family abuse, neglect and witnessing DVA. Similarly, those who had come from the most disadvantaged backgrounds, where family addictions and mental illness were common, were also more likely to mention feeling lonely from a young age. However, being able to name the feeling they had when they were young was not always easy for young women, and expressions of loneliness were implicit as well as explicit:

*I was quiet, I was timid and I was like wanting to please my parents, please everyone around me…I think when it happens like that especially when you’ve been in a hard… when you feel unwanted as a child *(Maya 19)

### Being silenced through labelling

Most of the young women in the current study described being negatively labelled throughout their lives and the consequences this had on their help-seeking behaviour. For example, young women commonly described how trying to tell others in the family about their abuse was met with disbelief and subsequently being identified as somehow ‘wrong’ within the family and their truth discredited. Labelling theory is concerned with the self-fulfilling effects of labelling and who it is that bestows the labelling—the social process by which the labelling is applied and its effect [52]. Consequently, the labels the family applied to the young women effectively blocked any further attempts at disclosure:

*Sometimes* [I talked to] *my brother but that was hard because sometimes he'd use it against me…Saying that I lied. That I’m a liar and I lied about it. *(Maya 19).

Being labelled as ‘*the black sheep’* in her family impacted on Lorraine’s help-seeking: ‘*It’s like if my own family don’t believe me and they know me better than anyone else, why would anyone else believe me?’.*

Outside of the family, Ellie described her perpetrator using labelling to take charge of the narrative around assault, effectively de-railing her help-seeking and leaving her more isolated:

*he* [man who sexually assaulted her when she was aged 12/13] *basically told his girlfriend that he’d cheated on her with me doing what he did and then I came out of this a homewrecker so then I couldn’t tell people what had actually happened, I didn’t really tell anyone.* (Ellie 25)

Being silenced through negative labelling at a young age reinforced the feelings of loneliness and isolation. A social process that continued at the next ecological level.

#### Relationship and community level response (Peers, Teachers, Schools, Church)

Outside of the home, the most important community level contact was schools. Participants often described acting out their distress or trauma from maltreatment, alongside more actively seeking help, whilst in school.

As reported in the literature [53, 54] teachers can be an important source of help and validation. However, most participants’ accounts of school responses to their behaviour were negative. Being negatively labelled within school was an interactional process creating a negative feedback loop; young women were labelled as ‘bad’ by peers, which was then reinforced by teachers (or the other way around) causing more ‘bad behaviour’. Participants described being bullied by peers and punished by the institution until the ‘bad’ label became a self-fulfilling prophecy.

*I didn’t drop out, I got booted out…Basically, I had issues with being bullied, because of who my parents were and what they’ve done… you know, “Your mum didn’t want you, you're a smack baby,” the usual. Which then progressed into my hair being cut, my blazer being set alight, whilst I was wearing it.*(Gemma 21)

The experience of being silenced through labelling happened more and to greater extremes for participants with disabilities, those from ethnic minority or socioeconomically disadvantaged backgrounds, or who were neurodiverse. Intersectionality compounded the experiences of being labelled. The concept of intersectionality [55] has widened over the years, but essentially describes how various forms of social stratification in society, such as race, class, gender, sexuality overlap and multiply inequities through the differential treatment of individuals.

Jackie clearly understood in retrospect the negative feedback loop in respect of institutional expectations around her educational abilities. At the time she felt highly visible, labelled and at the same time unheard because of her ethnicity, physical and learning disabilities:

*Being a black girl in an all-white school with disabilities, I had all the hallmarks to being bullied... It was blatant. If someone is calling you the ‘N’ word in the middle of the playground…the teachers said nothing. The teachers thought because I was in a lower grade at school that I would fail school. Which I did fail school because nobody was there to support me. *(Jackie 25)

Eleanor’s neurodiversity was mislabelled at primary school:

*The only thing that carried me through was in primary school I’d been taken out of my year for a year because my head teacher said I was retarded. Yes, and I had to have one-to-one lessons to learn to read, write, do maths. What happened is I went from a retard…*[to top of the class] (Eleanor 19)

Within school, some participants were referred to the school counsellor due to their acting out distress—labelled as behavioural problems. However, all accounts of school counselling services were negative. Ellie remembered her experiences being nullified, *‘I told her about being sexually abused when I was 12, she told me that that was just boys experimenting and exploring’*. Again, this response effectively silences the participant through invalidating their experiences of abuse and trauma.

Being silenced through denial was a persistent theme in our data. Participants’ school and peer level responses to their signs of trauma led to more loneliness. The feeling of being outcast or stigmatised by peers and teachers was clearly articulated by participants. Nina described the vicious circle of behaviour and response, culminating in entrenched isolation:

*I was bruised, I was scratched, bitten, everything, fag butts* [by abuser]*, that’s the reason why I did not take my clothes off in school. All the other girls did. I was standing there going, “Oh my God, it must only be me then.” Then you feel even more outcast and the school don’t want to be involved with you, and the kids don’t really want to be involved with you, because you’re different. Then, you’re isolated again there, where you’re not supposed to be.*

Feelings of isolation and loneliness were also due to the responses participants had received when they tried to seek help from services. Participants from the more advantaged backgrounds had less multi-victimisation and their accounts of loneliness and outsider feelings tended to be linked to mental health problems. This was exacerbated due to their mental health problems being denied by professionals, including school counsellors, but especially when peers invalidated mental ill health experiences:

*It did really, really, hurt to not be believed* [about aural and visual hallucinations] *. It really, really, did, but I just ended up becoming a bit more solitary after that.*(Nive 24)

However, those who had felt most isolated tended to be from more disadvantaged backgrounds, where often these feelings of being alone had carried on into adulthood, sometimes with a strong sense of self-reliance learned through adversity. When asked if she had received any good support in her life, Gemma responded: ‘*Not really. They all seem to fade away… I learnt to not rely on anybody but yourself ‘*

#### Community level response (Children’s Social Care, Police, Health Services)

The young women who had been recruited through frontline support organisations were more likely to have had early contact with social services. Descriptions of contact with social workers tended to be negative and the services provided were felt to be inconsistent. As found in previous research [45, 46] young people had often not felt heard or that their needs and wishes had been taken in to account. However, individual social workers *were* appreciated if they helped out the wider family.

Police were often involved with families when DVA and other maltreatment was present. Again, individual officers were praised. However, the police response generally reflected the accepted knowledge of the time, that only physical violence counted as abuse, as Leah described:

*Mum had to keep going to the police* [about abusive ex-husband] *The police did absolutely fuck all. They basically said, ‘until he physically hurts somebody, we're not doing anything’. So, you just felt like, "Well, what are you meant to do?" There's literally no one to help *(Leah 24)

The sense of isolation and loneliness was increased when there was misalignment of services. Young women offered group therapy for their maltreatment described situations where the treatments left them confused:

*At CAMHS* [Child and Adolescent Mental Health Services] *The whole family went to that, so a lot of shouting. It didn't help much. I didn't really know what the place was for. I was a troubled girl.*(Soraya 22)

#### *Cumulative loneliness* (life-course aspect of ecological model

When reflecting on their lives and making connections between past experiences and their own experiences of being with an abusive partner, participants linked entrenched feelings of loneliness with how easy it had been to end up in an abusive relationship:

*when I went home to my mum* [from care]*, I got into a relationship with Jaeden quite quickly, who is my daughter's dad. I still didn't know where my head was with things, really, and felt a lot of loneliness. I was trying to find a place of belonging, I guess, and I jumped into my relationship quite quickly, and then fell pregnant.*(Soraya 22)

Participants identified how loneliness had increased their vulnerability to further abuse and victimisation:

*the whole* [grooming] *stuff I think was, again, being lonely. Because, obviously, I got back with ex time, and time, and time, and time, again for the fact of being lonely. And then I got in with* [older girls, grooming] *because I thought, “Friends,” like.* (Nicky 19)

### Ways out of loneliness: finding a voice

Indications as to the positive factors or turning points in their lives were given in the young peoples’ descriptions surrounding ways out of being silenced and loneliness, primarily through being properly seen, heard and supported.

#### Relationship level

– Grandmother (‘Nan’)

Where ‘Nan’ could often be an important figure, a loving, consistent and – above all – safe person and place within difficult childhoods:

*my nan was my universe, so I lived with my nan… And I felt safe, for some years I felt really safe.* (Nina 23)

*My Nan was like my guardian angel, and she’d be there for me, no matter what… before she got unwell, she didn’t like the first stepdad at all. I would always go to my Nan and talk to her about it and she believed me *(Bec 22)

These findings align with evidence that having just one loving and consistent relationship with an adult can have a buffering effect on young people who have been maltreated [21, 47]. For the above young women, the grandparent was described as on their side and able to see the maltreatment within the family. Whilst all of these ‘nans’ had died when the participants were teenagers, a bereavement still felt keenly, their importance in providing a corrective foundation in chaotic lives was clear.

– Finding good friends and taking control

For those with friendships, the most helpful were considered the ones where there was open communication and full support when needed, especially in a crisis.

Escaping the home and school situation to further education and real, empathetic friends who truly heard worked as a revelation:

*it was the first time that somebody had taken me feeling… by the way I felt. Like it was a real problem. It's just because I had never had genuine friends like that.. I met people at uni and within a year, I was able to tell them so much stuff that I could never have told, or had never told, anyone else.*(Leah 24)

Having just one friend who came from a similar background and/or shared a similar experience can make a substantial difference:

*There’s one friend that has stuck through me, through in and out, and that’s X. She’d give the world for my kids, same as I would hers.* (Gemma 21)

Church had been a source of friendship and support outside of her dysfunctional family for Chloe:

*[I was] a bit lost. But I had my church friends. I never really felt a part of my family. I always felt different and detached from them. It was like watching someone else's home life, rather than mine, if that makes sense. I always used to wish that I was going to grow up and find out I was adopted, but that's not going to happen.*

Simply getting older and having more control over their lives, including financially, buffered the effects of early maltreatment. Leah had taken her experiences in her family and used them to achieve the independent life she wanted:

*I think I just had this absolute, utter determination to get myself the life I wanted. A good job, enough money that I would never be in a position where a man could hold my money.*

Over the life-course, with age and experience, participants described accepting and even welcoming what had once been stigmatising and isolating, for example:

*I was just always made to feel like an outsider, like something was always a bit weird about me. Which I still get, from people, now, but I embrace it now.* (Lily 22)

At the cultural level, changing perceptions of mental illness and identity politics in the last decade are likely to be reflected in how participants’ talk about themselves.

#### Community level

– Education, achievement

Education has the potential to be an extremely positive factor in ameliorating the impact of DV and abuse in the home – both at the time and investing in the futures of vulnerable children and adolescents:

*My little like safe haven- you know, wondering what the hell was going on at home. I came out with seven ‘A’s and nothing below a B. So, it worked, I suppose.* (Laughter)  (Lorraine 22)

A combination of academic achievement and good foster care had enabled Chloe to begin to fulfil her potential:

*school was alright, actually. I was a straight-A student. I excelled really well, academically. [when I went into foster care] I wasn't that smelly little girl in the corner, wearing the same clothes every day. So it got better. But then I just threw myself into my studies, so I didn't really care*

Achievements that may seem minor to others can and do have an impact on participants feelings of self-worth and ability to envisage another future. Tutors who are prepared to put in the extra effort and support for young people who have been labelled ‘difficult’ are extremely important in this future. This was particularly true for those from disadvantaged backgrounds, such as Gemma:

*I’ve fought tooth and nail to be able to get onto my child-minding course. But I was told repeatedly four times over the space of two years because of my past and trouble I’ve been in as a child I wouldn’t be able to work with kids. So I was slowly dropping out of college, but luckily enough I managed to scrape through by the skin of my teeth because my tutor understood me, she understood my point. She helped me go through the assessment for my learning disabilities that the other schools hadn’t listened to.*

Teachers hold a unique position to be a consistent, observant and supportive adult outside of the family unit. Participants described responding positively to being seen and heard by teachers and trust was very important in the teacher/pupil relationship where trust within the family and elsewhere had been broken; in Chloe’s case she had felt able to disclose about the neglect and abuse in her home due to addictions, and being raped by her half-brother, ‘*my tutor was amazing. She was the one that I told…She was just really nice. She was so sweet’.*

Finally, there were descriptions of positive police responses to help seeking behaviour, of being heard and taken seriously, such as Chloe’s, ‘*the police got involved, and that's when they actually started- "Right, we need to start paying attention to what she's saying, rather than a young girl lashing out*’. 

Positive descriptions of interactions with the police increased over the life-course of the participants’ accounts, which was likely the result of changing police policy around domestic abuse, reflecting the shifting societal/cultural knowledge influenced by activism, research and journalism within the sector.

### Quantitative findings

Two thousand one hundred twenty-seven women and 1145 men were included in our analysis. Characteristics of these participants, and how they compare with the original ALSPAC cohort are described in Supplementary Table 1. Study cohort members were less likely than those of the full ALSPAC cohort to have mothers who had already borne children, who had a degree, or be in high social class positions (based on highest social class of the mother and father); they were also less likely to be part of single parent households, or be a Person of Colour, but were more likely to report not being 100% heterosexual. For all of the above variables, they were less likely to have missing data.

Child maltreatment was associated with a twofold higher chance of both female and male 14 year-olds reporting that school was a place they felt lonely (OR for women: 1.90, 95% CI 1.31 to 2.77; OR for men 2.35, 95% CI 1.24 to 4.43) (Table 2). In males, there was also an association between child maltreatment and loneliness within peer group (feeling that your friends did not understand you, at age 13); OR 2.35, 95% CI 1.24 to 4.43. This association was not seen in females (1.05, 0.50 to 2.19). In females, there was weak evidence of an association between child maltreatment and overall loneliness in the past two weeks at age 13 (OR 1.66, 95% CI 1.00 to 2.76); this association was not seen in males (1.39, 0.60 to 3.25). Adjustment for covariates did not alter these associations.

**Table 2:** Associations between child maltreatment and loneliness during adolescence. *N* = 2127 females and 1145 males, using imputed data

	**Odds ratio for the association between child maltreatment and loneliness during adolescence (95% confidence interval)**					
	***Females***			***Males***		
	*Loneliness at school at age 14*	*Loneliness within peer group at age 13*	*Overall loneliness at age 13*	*Loneliness at school at age 14*	*Loneliness within peer group at age 13*	*Overall loneliness at age 13*
Maltreatment* between 0–12 years (unadjusted)	1.90 (1.31 to 2.77) *P* = 0.001	1.05 (0.50 to 2.19) *P* = 0.90	1.66 (1.00 to 2.76) *P* = 0.05	2.35 (1.24 to 4.43) *P* = 0.01	2.87 (1.20 to 6.85) *P* = 0.02	1.39 (0.60 to 3.25) *P* = 0.45
Maltreatment* between 0–12 years (adjusted**)	2.03 (1.38 to 2.97) *P* < 0.001	1.05 (0.51 to 2.16) *P* = 0.90	2.67 (1.00 to 2.79) *P* = 0.06	2.33 (1.23 to 4.44) *P* = 0.01	3.05 (1.27 to 7.32) *P* = 0.01	1.33 (0.57 to 3.13) *P* = 0.51

^*^Maltreatment = emotional abuse, emotional neglect, physical abuse, sexual abuse, domestic violence (violence between ‘parents’), bullying

^*^^*^Adjusted models include the following covariates for females: maternal age at delivery, maternal parity at delivery, maternal education, whether the child lives in a single parent household. For males, the same set of covariates was used apart from single parent household, which could not be included due to perfect prediction in one or more of the imputed datasets

Child maltreatment was associated with an increased risk of IPVA in both females and males (Table 3). Loneliness at school at age 14 was associated with IPVA in females (OR 1.63, 95% CI 1.13 to 2.35) and males (OR 1.76, 95% CI 0.91 to 3.40). ORs for the associations of loneliness within peer group and overall in the past two weeks with IPVA were positive for females, but with very wide confidence intervals, but there was no evidence of associations between these measures of loneliness and higher risk of IPVA in males.

**Table 3:** Associations of maltreatment and loneliness with IPVA (2127 females, 1145 males, imputed data)

	**Odds ratio for the association of child maltreatment or loneliness in adolescence with IPVA in young adult relationships between 18–21 years old (95% confidence interval)**			
	**Females (unadjusted)**	Females (adjusted**)	Males (unadjusted**)	Males (adjusted**)
Child maltreatment* 0–12 years	1.24 (1.01 to 1.52) *P* = 0.04	1.22 (0.99 to 1.50) *P* = 0.06	1.39 (1.03 to 1.87) *P* = 0.03	1.37 (1.01 to 1.84) *P* = 0.04
*Loneliness at school at age 14*	1.63 (1.13 to 2.35) *P* = 0.01	1.64 (1.14 to 2.37) *P* = 0.01	1.76 (0.91 to 3.40) *P* = 0.10	1.68 (0.86 to 3.26) *P* = 0.13
*Loneliness within peer group at age 13*	1.55 (0.80 to 3.01) *P* = 0.20	1.53 (0.79 to 2.98) *P* = 0.21	0.80 (0.34 to 1.94) *P* = 0.65	0.81 (0.34 to 1.94) *P* = 0.65
*Overall loneliness at age 13*	1.40 (0.87 to 2.28) *P* = 0.16	1.41 (0.87 to 2.29) *P* = 0.16	1.04 (0.46 to 2.35) *P* = 0.92	0.99 (0.44 to 2.26) *P* = 0.99

^*^Maltreatment = emotional abuse, emotional neglect, physical abuse, sexual abuse, domestic violence (violence between ‘parents’), bullying

^**^Adjusted models include the following covariates for females: maternal age at delivery, maternal parity at delivery, maternal education, whether the child lives in a single parent household. For males, the same set of covariates was used apart from single parent household, which could not be included due to perfect prediction in one or more of the imputed datasets

There was some evidence that loneliness at school mediated a small proportion of the association between child maltreatment and IPVA in females and males (shown by the direct effects being smaller than the total effects in Table 4), but the direct effect was still very similar to the total effect (e.g. in females, 1.27 for the total effect, 1.24 after accounting for all three loneliness measures), suggesting the vast majority of the association between child maltreatment and IPVA is not mediated through loneliness at school. For the other measures of loneliness, total and direct effects were very similar, suggesting these are not important mediators of the association between child maltreatment and IPVA.

**Table 4:** Mediation of association between maltreatment and IPVA, by loneliness (2127 females, 1145 males, imputed data*)

	**Odds ratio for the association of child maltreatment with IPVA in young adult relationships between 18–21 years old; before (total effect) and after (direct effects) accounting for mediation by loneliness (95% confidence interval)**	
	Females (adjusted***)	Males (adjusted***)
Total effect of child maltreatment** on IPVA	1.27 (1.05 to 1.54) *P* = 0.06	1.46 (1.09 to 1.94) *P* = 0.01
Direct effects; i.e. the association between child maltreatment* and IPVA after accounting for****:		
*Loneliness at school at age 14*	1.27 (1.02 to 1.49) *P* = 0.03	1.42 (1.07 to 1.89) *P* = 0.02
*Loneliness within peer group at age 13*	1.26 (1.05 to 1.52) *P* = 0.02	1.47 (1.10 to 1.95) *P* = 0.01
*Overall loneliness at age 13*	1.26 (1.04 to 1.52) *P* = 0.02	1.46 (1.10 to 1.93) *P* = 0.01
*All three loneliness measures*	1.24 (1.03 to 1.50) *P* = 0.03	1.43 (1.07 to 1.91) *P* = 0.02

^*^Pooled beta coefficients and standard errors were derived across imputed datasets using Rubin’s rules. Mean *p*-values across the imputed datasets are reported

^**^Maltreatment = emotional abuse, emotional neglect, physical abuse, sexual abuse, domestic violence (violence between ‘parents’), bullying

^***^ Adjusted models include the following covariates for females: maternal age at delivery, maternal parity at delivery, maternal education, whether the child lives in a single parent household. For males, the same set of covariates was used apart from single parent household, which could not be included due to perfect prediction in one or more of the imputed datasets

^****^The ‘natural direct effect’

## Discussion

In interviews with young women, how individuals and institutions responded to young peoples’ help-seeking around maltreatment and wider forms of disadvantage was essential to the well-being and consequent behaviour of respondents.

Being silenced acted as a social determinant not only of mental and physical health and wellbeing but operates to further isolate young people leading to intense loneliness, increasing the risk of further victimisation/maltreatment for children and young people.

Most interview participants had been silenced in a combination of ways; through by not being listened to, by being negatively labelled and due to poor or misaligned professional services that did not address, or in some cases denied, the experiences of the young people involved.

Silencing happened at all ecological levels from family and peers to community and institutions including schools, police, health and other professional services. The experience of not being heard was compounded by structural factors: the class, ethnicity, sexuality, gender identity, disability and neurodiversity of young people often played a part in how people at the individual, interaction level responded to participants. This was not necessarily conscious; systemic, cultural biases at the societal level can be a powerful disincentive to disclosing abuse.

As a result, participants reported lifelong feelings of loneliness and/or of being an outsider. This acted as a primary mechanism in a negative feedback loop/pathway: feeling or being ‘othered’ can increase vulnerability to more maltreatment leading to increased ‘othering’ and loneliness, leading to increased vulnerability to more maltreatment.

Quantitative analyses in the ALSPAC cohort, exploring this pathway from early childhood maltreatment and trauma to IPVA through loneliness, showed that loneliness mediates an important minority of the pathway between early years victimisation and IPVA. Among interviewee accounts, this effect was stronger for those who had been multi-victimised, and was compounded by compounded by disadvantage, ethnicity and disability.

A number of studies have found that the reactions of professionals to young people disclosing maltreatment were perceived as unhelpful; such as not being believed, or no action being taken [56, 57]. Our findings on silence support those of Mackenzie’s [58] where they a ‘*operate to undermine an entitlement to be heard and to obscure the ways in which abuse (past or present) acts as a social determinant of mental and physical health*.’ [2019: Pg 4].

Where the current findings add to the theories of help-seeking, is by highlighting the social process of labelling as part of the silencing mechanism that ‘others’ the participants who are already vulnerable due to negative family experiences and maltreatment of all kinds.

Participants’ accounts have a lot in common with labelling and stigmatising described in education by Thomas (1997). Labels help create and organize the options available to students. Once constructed as a victim, stigmatised with negative labels, and rejected by peers, studies show it is almost impossible for students to change and improve their situation [59]. Even when external victimisation ends, an internalised version can continue and cause psychosocial problems for many years affecting the victims’ relationship with others.

However, these micro interactions do not operate in a vacuum; biases that are present at the societal and structural level are enacted by family, peers, teachers, health, police and other professionals at the relationship and community level. Again, they may not be explicit biases—but they are biases nonetheless and are risks that can lead to further isolation and vulnerability to maltreatment such as peer abuse and IPVA.

Whilst feelings of loneliness and isolation are one of the consistent consequences reported by women in abusive intimate relationship and children who have been sexually abused [9], the role of loneliness in increasing vulnerability to further maltreatment and IPVA has not been fully described before. One other small study of three young women’s perceptions of IPVA touched briefly on their vulnerability to IPV being partly to do with feeling disconnected [60] a related, if not analogous, emotion. A quantitative study [51] made the case for loneliness as a mediator between childhood abuse, shame and health. The current study addresses, in part, the authors’ concern that much is still unknown about the role of other people's responses in the development of shame and loneliness in those who were not sufficiently protected in childhood. Our findings show how silencing and labelling by family and community of childhood maltreament and trauma causes loneliness and increasing vulnerability to IPVA.

Whilst we recognise that this is not the sole responsibility of schools and teachers, they do nevertheless play an important role in children and young adults’ lives as evidenced in how participants spoke about their experiences. How teachers responded to signs and signals of distress was often a potential turning point for vulnerable, lonely children and adolescents.

Whilst not being heard acted as a barrier to support, examples of good support involved being believed and fully seen and listened to by one trusted family member or friends and/or a professional adult or service, which could be the first step in getting help [21, 25].

Being given prompt and *appropriate* help can aid young people in understanding their situation and begin ‘recovery’; addressing the underlying causes of mental health symptoms and acting out behaviour with the correct treatment.

Many participants had been multi-victimised and had negative experiences when trying to seek help that had left them lonely and vulnerable. As reported in other studies, ‘dual exposure’, living with parental domestic violence as well as other forms of child maltreatment, increases the odds that a young person will experience intimate partner violence [5].

However, and it is a large ‘however’, whilst there were disadvantaged participants who had experienced multiple adversity *they kept going*—sometimes in the face of seemingly insurmountable barriers. They wrestled control back of their lives by fighting for their education whether a GCSE, an NVQ or a degree. They had plans for the future, for their children (if they had them), for their career, for making a difference.

Having the opportunity to achieve in education was essential to feelings of self-worth and future expectations and prospects. This was particularly true for disadvantaged participants where achievements gave them choice and consequent financial freedom and, therefore, the possibility of breaking the pattern of intergenerational transmission of violence and abuse.

Having experiences validated and being supported, whether by a family member, peer or member of a community, profession or institution, ameliorated the impact of the maltreatment/loneliness loop.

Listening to young womens’ life experiences prior to IPVA helps us to better understand what helped them at a relational and community level (and not just what hindered). The strengths-based approach to violence prevention makes clear that what helps young adults may not seem to be directly to do with violence or health [15]. A wider, ‘cross-cutting’ approach is needed, rather than professions and disciplines working in isolation, in directing prevention and intervention and building strength in any community. The current findings build on this approach by indicating what different individuals and groups want to achieve and what would help them to achieve this. In this case by highlighting the strength of education; making it easier for those who are most vulnerable to stay in school or college and support them towards their goals. It is important that abuse is addressed in these extra-familial domains to help those affected. Resilience is not an individual trait but the work of everyone around the young person; their family, teachers, services/community and structures [61].

It is important to clarify that the unjust way the participants were treated due to their socioeconomic group, ethnicity, gender, sexuality, disability or neurodiversity at relationship and community levels were directly linked to the laws, systems and inequalities at the societal level. The participants who were most disadvantaged had continued to be so, their situation intensified by social exclusion and poverty.

Overall, there were fewer accounts of positive factors compared to previous work, possibly as an artefact of recruiting people through frontline services who were more likely to have been multi-victimised. There was some evidence that participants from more advantaged backgrounds had more access to resources to deal with their maltreatment e.g. private counselling, greater social networks and social capital. Nevertheless, for lower and higher socio-economic participants, the ways in and out of the loneliness loop were similar, at all ecological levels.

### Strengths and limitations

The sensitive nature of the research ensured that recruiting could only take place through organisations or via groups where participants could access support after their interview if necessary. This resulted in participants who were more likely to have been ‘multi-victimised’ than a more general population sample and nearly all participants had some experience of some form of counselling.

The original aim of the study was to recruit and interview young male and female adults. Despite best efforts, we did not manage to engage support organisations specifically for men to help us recruit, although one young man was recruited and interviewed through a frontline organisation. Consequently, our findings can only describe the data from the perspectives of the young women.

Nevertheless, the intention was to sample as wide a range of young adults as possible and recruitment included those from: advantaged and disadvantaged backgrounds; a range of ethnic minority backgrounds and sexualities, with and without higher education or employment, children or no children, with or without disabilities and atypical or neurodiverse. A strength of the study was in reaching data saturation in this varied sample in the themes presented, adding to the validity of the findings. The use of LHCs also directly addressed problems of recall bias by evoking memories ‘underneath’ the usual narratives given.

When considering the different strengths of the qualitative and quantitative findings, it is likely that the stronger outcome for loneliness in the interview data was associated with the majority of participants having a more economically disadvantaged and multi-victimised background than that of the ALSPAC cohort. The different findings may also be explained in part by the different framing of ‘loneliness’: ALSPAC participants were asked at specific timepoints and in surveys about feeling lonely; qualitative participants were not asked about feeling lonely – they spoke about it spontaneously throughout their interviews in the context of their life history. Further research investigating loneliness as a mechanism or risk for IPVA within and between different socially determined groups would build on this finding.

### Implications

Only relatively recently has there been a sustained UK public health focus on IPVA in young peoples’ relationships [44, 62, 63]. IPVA is a complex and multifaceted issue and young people require differential levels of support depending on the risks they encounter. There is a need to recognise how wider structural inequalities intersect with IPVA perpetration, necessitating the need for population-based prevention programmes [1].

Our findings show that participants tried to get help for maltreatment prior to IPVA in their childhood and adolescence, they have agency; but it is essential that young adults – especially those most at risk of isolation and loneliness and therefore more vulnerable—are listened to and believed. Good practice needs to focus on early-intervention, service-user led prevention strategies.

Pre-existing vulnerabilities need to be addressed by services and initial support should be needs-led. It is important to consider the cross-cultural aspects of children and young adults’ experience and provide services that are multidimensional and respond to different cultures and experiences. For example, how distress or loneliness manifests will vary between and within all cultural groups. It is important to consider both the complexity of experiences *and* cumulative effects of adversity.

For young people, the school environment and relationship education is key. However, the importance of interventions outside of school in community groups and settings, or in clinical contexts should also be considered for a fully informed approach at all levels of young adult interactions. There are many access points of intervention for young people experiencing maltreatment which may help break the inter-generational cycle of violence and victimisation.

Care needs to be taken to ensure society and institutions address inequities in responses to children and adolescents reporting maltreatment. The current findings support research into the importance of active listening and being believed (in children and adolescents), not just in disclosure of victimisation and multi-victimisation itself, but in breaking the transmission of intergenerational transmission of violence and victimisation.

## Supplementary Information

**Additional file 1: Supplementary Table 1. **Distribution of characteristics in study cohort (*n*=2127 females, 1145 males) v full ALSPAC cohort* (*n*= 7,347 females, 7,688 males).

## Data Availability

The qualitative datasets generated and analysed during the current study are not publicly available due to the highly sensitive nature of the research and the need for strict anonymity and confidentiality for the participants involved. The informed consent obtained from ALSPAC participants does not allow the data to be made freely available through any third party maintained public repository. However, data used for this submission can be made available on request to the ALSPAC Executive. The ALSPAC data management plan describes in detail the policy regarding data sharing, which is through a system of managed open access. Full instructions for applying for data access can be found here: http://www.bristol.ac.uk/alspac/researchers/access/. The ALSPAC study website contains details of all the data that are available (http://www.bristol.ac.uk/alspac/researchers/our-data/).
