# Supplementary material for: Being silenced, loneliness and being heard: understanding pathways to intimate partner violence & abuse in young adults. a mixed-methods study
Source: BMC Public Health. 2022 Aug 17;22:1562. doi: 10.1186/s12889-022-13990-4 (PMC9381391; doi:10.1186/s12889-022-13990-4)

**Supplementary Box S1: Details of questionnaire used to assess IPVA**

The full questionnaire is available here: http://www.bristol.ac.uk/media-library/sites/alspac/documents/questionnaires/20131213%20YP%20Your%20Life%20Now%20Questionnaire.pdf


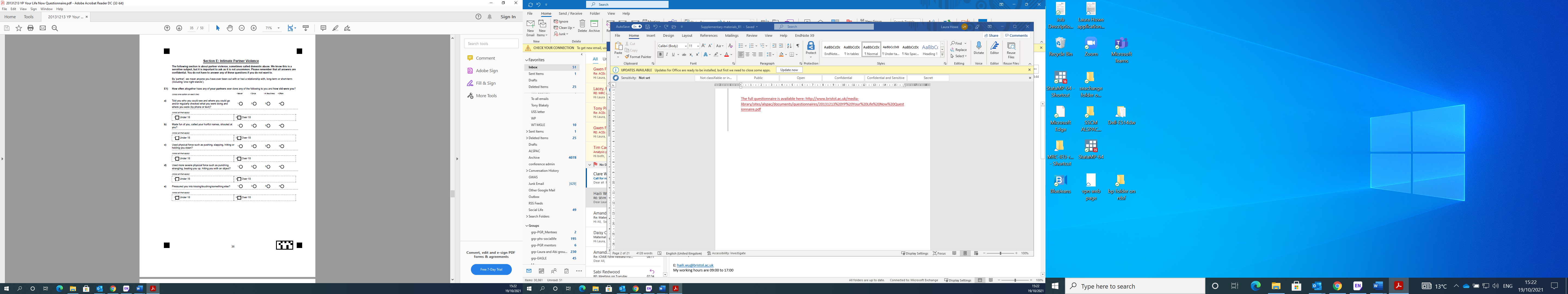


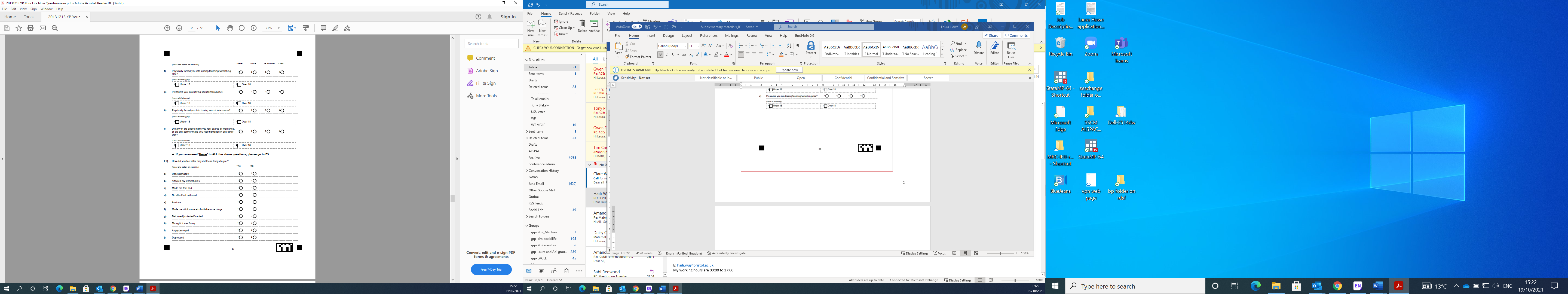


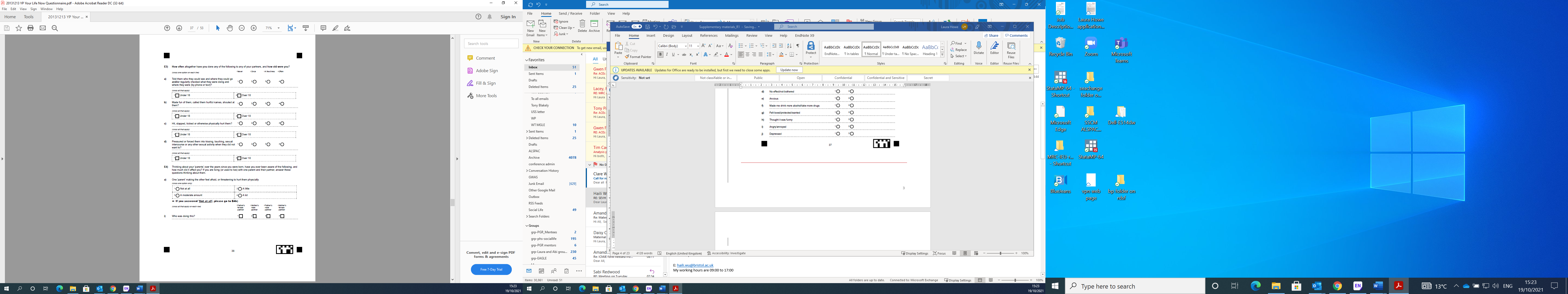

Supplement: Supplementary file 1 — Additional file 1: Supplementary Table 1. Distribution of characteristics in study cohort (n=2127 females, 1145 males) v full ALSPAC cohort* (n= 7,347 females, 7,688 males). [file 12889_2022_13990_MOESM1_ESM.docx]
